# Do Environmental Regulations Promote or Inhibit Cities’ Innovation Capacity? Evidence from China

**DOI:** 10.3390/ijerph192416993

**Published:** 2022-12-17

**Authors:** Xiaowen Zeng, Ming Jin, Shuang Pan

**Affiliations:** 1 The Faculty of Economics, Guangdong University of Finance & Economics, Guangzhou 510320, China; 2 School of Economics, Zhejiang University of Finance and Economics, Hangzhou 310018, China; 3 School of Accountancy, Jiangxi University of Finance and Economics, Nanchang 330013, China

**Keywords:** environmental regulation, urban innovation, mediating effect

## Abstract

The “Porter Hypothesis” proposes that appropriate environmental regulations would promote firm innovation. This study aims to build a theoretical model for illustrating the impact and mechanism of environmental regulation on urban innovation through a panel of 281 Chinese prefecture-level cities during 2003–2016. The results indicated that an increase in environmental regulation markedly suppressed the innovative capacity of Chinese cities during the sample period. This inhibitory effect is primarily transmitted through two mediating variables: lower regional fiscal revenue and reduced manufacturing output. Moreover, improved regional economic development level helps generate positive incentives for environmental regulation and mitigate its inhibitions to innovation. Environmental regulation and urban innovation might have a non-linear U-shape relation, with the former helping improve urban innovation capacity upon reaching a particular level.

## 1. Introduction

Recently, environmental problems have become more and more serious in the world. Global climate changes, acid rain, water pollution, air pollution and other types of environmental degradation are becoming increasingly common. China is not exempt from such environmental problems either. Relying on massive inputs of production factors from the reform and opening to foreign investment in 1978, the Chinese economy has achieved remarkable success, growing at over 9% per year from 1978 to 2021. It is undeniable that while the extensive growth approach has brought huge economic gains, it has also caused severe environmental pollution. Recently, China has been deepening sustainable development strategies and promoting a comprehensive green transformation of its development. This has led to the introduction of numerous environmental policies and regulations throughout China to prevent and control environmental risks tightly. Environmental quality keeps improving with the implementation of “closure, suspension, merger, or shifting to different line of production” of high-energy-consuming and high-polluting enterprises. However, this inevitably has a significant impact on regional economic development. When governments implement stringent environmental regulations and low-carbon policies, enterprises face various risks when transitioning to more environmental practices due to uncertainty about the future and increased operating costs. Moreover, local development may also be affected. Thus, the study of environmental regulation is essential to regional development.

Whether environmental protection and economic growth can be achieved concurrently has been extensively debated in both domestic and international academic circles. It is suggested that environmental regulation would aggravate the financial burden on firms and thus reduce their international competitiveness [1] and affect firms’ total factor growth rate [2]. However, several researchers proposed that environmental regulation can have positive effects. For example, Porter argued that appropriate environmental regulation stimulated innovation and increased the competitiveness of firms, despite raising their costs, which is subsequently summarised as the “Porter hypothesis” [3]. However, there is still no clear consensus on whether this view is applicable to China. When firms face environmental regulation, a more effective solution is to reduce corporate pollution and increase revenue through innovative means. Some thus argued that incentive-based environmental regulation in China could enhance corporate innovation [4]. However, firms face transition risks such as lower revenues and higher costs under environmental regulations. Additionally, research and development (R&D) activities are inherently risky corporate behaviours, and enterprises may also lower R&D expenditures due to difficulty in providing sufficient funding or reducing corporate risk [5]. Moreover, poorer economic development can cause environmental regulations to inhibit innovation [6]. 

According to the existing studies, the change in environmental regulation intensity will have a significant impact on the innovation behaviour of enterprises, and the overall regional innovation level is closely related to the innovation ability of local enterprises. Therefore, it is not difficult to guess that environmental regulation is likely to have a corresponding impact on regional innovation. Although some scholars have analysed the micro-impact of environmental regulation from the perspective of enterprise behaviour, they mainly focus on listed companies or enterprises above a designated size, which will bring sample selection bias and make it difficult to identify the impact of environmental regulation. In addition, the relationship between urban innovation and environmental regulation will be more complex. On the one hand, local enterprises will be forced to transform due to stricter environmental regulations, which will significantly increase regional innovation. On the other hand, when the intensity of environmental regulations has been enhanced, polluting enterprises may shut down or reduce production in order to reduce the cost of pollution control, which will lead to the reduction of enterprise innovation activities and the decline of regional innovation ability. These changes are difficult to observe only through enterprise-level data. Therefore, this paper believes that the empirical study on the panel data of 281 prefecture-level cities is helpful to further explore the micro-impact mechanism of environmental regulation policies on regional innovation ability. Based on the city as an innovation carrier, this paper made several contributions: (1) Being different from the Porter hypothesis, this study finds that an increase in environmental regulation significantly suppressed cities’ innovation capacity. At the same time, this paper adopts urban innovation data at the prefecture level, which can avoid sample selection bias. It can reflect the overall impact of environmental regulation policies on urban innovation ability and provide research support for investigating the spillover effect of environmental regulation policies. (2) This paper innovatively explores how Chinese environmental regulation inhibits urban innovation capacity. On the one hand, with the increase of environmental regulations, the decline in business efficiency of enterprises in the short term will bring a significant decline in local fiscal revenue, and the local government may be forced to reduce the subsidy support for enterprises’ innovation, which will bring a restraining effect on urban innovation ability. On the other hand, since most Chinese manufacturing enterprises are still in the transition stage from extensive production to efficient production, blindly strengthening environmental regulation intensity is easy to lead to the decline of local manufacturing output and obstacles to technology research, thus restricting the improvement of urban innovation ability. (3) This paper conducts a variety of robustness tests on urban heterogeneity, environmental regulation policy categories, green technology innovation and other aspects and draws a series of new conclusions: in areas with poor economic development and less fixed investment, environmental regulation has a more significant inhibiting effect on urban innovation capability. In non-knowledge-intensive cities, the impact of environmental regulation is significantly negative, while in knowledge-intensive cities, the impact is not significant. Both market-oriented and command-and-control environmental regulation policies have a significant inhibitory effect on urban innovation ability in the sample period. (4) This study demonstrated the specific non-linear association between environmental regulation and urban innovation, which lends empirical proof for the Porter hypothesis at the meso-level. At the same time, it is clearly pointed out that China is still in a painful period of transition from extensive development to high-quality development, and the negative effects of environmental regulation policies will temporarily outweigh the positive effects. It is necessary to pay attention to the negative spillover effects of environmental regulation on technological innovation. This manuscript is organised below: Chapter 2 reviewes the relevant works. Chapter 3 introduces the theory model derivation. Chapter 4 provides the empirical test analysis and further research. The final section offers conclusions and policy advice.

## 2. Literature Review

### 2.1. Environmental Regulation and Urban Innovation

Environmental protection and economic growth are enduring topics of academic debate, and the loss of economic benefits due to environmental protection has long been thought-provoking for numerous scholars. Environmental regulation by governments can be a valuable motivator for firms to implement environmental initiatives [7]. Magat found that environmental regulation can influence firm innovation, but the effects vary depending on the environmental regulation type [8]. Scholars also argued that environmental regulation could lower the productivity level and growth rate of industries [1]. Porter countered that environmental regulation does not necessarily result in economic losses, suggesting that appropriate environmental regulation may spur ‘innovation compensation’ to enhance firm innovation [3]. Subsequently, Porter and Linde specified that innovation may occur when organisations attempt to increase the environmental efficacy in resource use, thus helping the production processes and product quality improvement [9].

### 2.2. Environmental Regulation and Economic Development

At present, China continues to implement low-carbon environmental policies, accompanied by increased environmental regulation in various regions. Many scholars have begun to discuss the link between environmental regulation and economic development. Several academics argued that environmental regulations caused the operating costs to increase and productivity to decline to some extent. For example, Guo discovered that environmental regulations fail to enhance green growth straightforwardly [10]. Yuan verified that environmental regulation reduces R&D investment over a long period utilizing panel data on Chinese manufacturing [5]. He utilised a Chinese water quality monitoring system and found that local governments implement more rigorous environmental criteria for enterprises upstream of monitoring sites [2]. Wu identified a significant U-shape relationship between environmental regulation and the green factor productivity of China’s energy sector [11]. In contrast, Du argued that poor economic development can cause environmental regulation to stifle green technological innovation [6]. However, several academics hold the view that environmental regulation could prompt technological innovation and productivity improvement, especially in clean production industries [12]. Fu and Li found that environmental regulations promote innovation while increasing firms’ costs, thereby improving their competitiveness [13]. Pan found that as market-based environmental regulations progressively enhance the energy efficiency, technological innovation will also be impacted by these regulations [4]. Additionally, market-based and voluntary environmental regulations possess a greater incentive effect on business innovation than that from command/control-based environmental regulations. The above thereby validated “Porter’s hypothesis”.

### 2.3. Environmental Regulation and Industries’ Innovation Capacity

Concurrently, numerous researchers addressed the connection between environmental regulation and industries’ innovation capacity in the Chinese manufacturing sector. Yuan conducted a study on Chinese manufacturing firms and determined that environmental regulation decreases their R&D investment [5]. Studies based on industry classification have apparent advantages: as performance measures tend to be consistent across industries, industry classification studies specify environmental regulation’s impact on industries with varying development and pollution levels. Thus, their findings are more credible. However, there are limitations in that multiple industries exist in the same region. Thus, local governments need to consider multiple industries when making decisions, as different types of industries are affected by environmental regulations to different degrees. Hence, the regional role of environmental regulation also requires investigation. For companies of the same region environmentally regulated with similar degrees, an examination of regional environmental regulation has greater potential to highlight its regional role and be more informative for the implementation of local governmental policy.

Additionally, other scholars have conducted region-based studies about environmental regulation and firm innovation. Nie found that environmental regulation fostered innovation among less developed regions of western China and demonstrated the applicability of Porter’s hypothesis in less developed regions of developing countries [14]. Li revealed that environmental regulations did not significantly affect the efficiency of urban science and technology innovation in Xi’an, suggesting the inapplicability of the Porter hypothesis in Xi’an [15].

In summary, focusing on Porter’s hypothesis, the established research mainly examined the influence of environmental regulations on innovation at a micro level. However, research at the prefecture level has been insufficient and thus needs to be expanded. Concurrently, studies at the regional level centred on the correlation of environmental regulation to regional economic development but have been unable to identify and validate the mechanisms of its impact. Thus, this study examined the effects and transmission mechanisms of environmental regulation towards urban innovation using meso-level data to serve as a reference for setting regional environmental regulation policies.

## 3. Mathematical Model Analysis

The ability of cities to innovate depends on economic support and talent development. Many firms are at the forefront of urban innovation. “Porter’s hypothesis” suggests that there exists a possible non-linear relationship between environmental regulation and firm innovation, i.e., environmental regulation may inhibit firm innovation in the short term but increase firm innovation and competitiveness in the long run. Similarly, this study argues that environmental regulation may ultimately have a corresponding impact on the innovation capacity of urban areas by influencing firms’ production and business behaviour. Thus, the following mathematical model was derived by drawing on the research method of Zhang et al. (2011) [16]. We mainly extended Zhang’s theoretical model to the city level and shifted the research perspective to the field of urban innovation ability.

Assumptions: Manufacturers conduct production activities in a perfectly competitive product and factor market; as production expands, the pollution generated by the manufacturers increases accordingly.

Let the vendor’s revenue function be:

$$R=P*A\left(K_{A}\right)f\left(K_{P}\right)$$

where P denotes the price of products, $K_{A}$ denotes the capital input used in production for technological innovation, and $K_{P}$ represents the capital input to the daily production of the firm. $A\left(K_{A}\right)$ represents the level of technological innovation in production, and $f\left(K_{P}\right)$ represents the level of output at the given level of innovation.

Then, the output function of the manufacturer can be expressed as $F=A\left(K_{A}\right)f\left(K_{P}\right)=Af$. Here, it is assumed that the level of innovation in production with technological innovation is Hicks neutral.

As manufacturers produce emissions in the production process and pollution has negative externalities, the government will regulate manufacturers’ pollution behaviour by specifying a level of pollution, i.e., environmental regulation (ERS). Existing research suggests that, under government environmental regulation, manufacturers may first reduce the level of pollution emissions from their production process through technological innovation in their production processes to achieve a lower level of pollution; then, manufacturers may increase their R&D investment to increase their output level. Although this will lead to more pollution emissions, firms can increase their pollution control expenditure in response to environmental regulation, benefiting from the increased scale of output and profits. It can be deduced that manufacturers’ technological innovation is related to their own production technology A and pollution control technology E. Based on this, the present study argues that urban innovation in the context of environmental regulation can also be composed of two parts: regional productive technological innovation $\left(CI_{A}\right)$ and the pollution control technology innovation induced by environmental regulation ($CI_{E}$). This study further assumes that the urban innovation function is separable; thus, $CI=CI_{A}+CI_{E}$, which satisfies $CI^{\prime }=\left(A,\cdot \right)>0,CI^{\prime }=\left(\cdot ,E\right)>0$.

Additionally, the manufacturer’s emission function is assumed to be W=(F,E), which is a function of the level of output and the pollution control technology. This function has the following properties:

First, pollution emissions increase with the increase in the scale of output, i.e., $W^{\prime }=\left(F,\cdot \right)>0$. Second, pollution emissions decrease as pollution control technology improves, i.e., $W^{\prime }=\left(\cdot ,E\right)>0$. Apparently, *E* is positively correlated with the intensity of environmental regulation; it is believed that an increase in the intensity of environmental regulation will be followed by an increase in technological innovation in pollution treatment.

Assuming that the portion of firms’ total output devoted to pollution control α denotes the intensity coefficient of environmental regulation, where α represents a real number between 0 and 1, then $\alpha A\left(K_{A}\right)f\left(K_{P}\right)=E$. Thus, the final profit function of the manufacturer is as follows: $\pi =P\left[A\left(K_{A}\right)f\left(K_{P}\right)-\alpha A\left(K_{A}\right)f\left(K_{P}\right)\right]$. The constraint under which the manufacturer produces is then as follows:

$ERS=W\left[A\left(K_{A}\right)f\left(K_{P}\right),\;\alpha A\left(K_{A}\right)f\left(K_{P}\right)\right]$, i.e., the pollution emissions are equal to the environmental regulation.

Constructing Lagrangian functions:

$$L=P\left[A\left(K_{A}\right)f\left(K_{P}\right)-\alpha A\left(K_{A}\right)f\left(K_{P}\right)\right]+\lambda \left\{W\left[A\left(K_{A}\right)f\left(K_{P}\right),\;\alpha A\left(K_{A}\right)f\left(K_{P}\right)\right]-ERS\right\}$$

The first-order optimality condition for the manufacturer is solved by the Lagrangian function as:

(1) $$P\left(1-\alpha \right)A^{\prime }f+\lambda \frac{\partial W[A\left(K_{A}\right)f\left(K_{P}\right),\;\alpha A\left(K_{A}\right)f\left(K_{P}\right)}{\partial K_{A}}=0$$

(2) $$P\left(1-\alpha \right)Af^{\prime }+\lambda \frac{\partial W[A\left(K_{A}\right)f\left(K_{P}\right),\;\alpha A\left(K_{A}\right)f\left(K_{P}\right)}{\partial K_{P}}=0$$

(3) $$-PAf+\lambda \frac{\partial W[A\left(K_{A}\right)f\left(K_{P}\right),\;\alpha A\left(K_{A}\right)f\left(K_{P}\right)}{\partial \alpha }=0$$

From Equation (3), we obtained

(4) P=λ⋅∂W/∂E

Bringing Equation (4) into Equation (1) yielded the following:

(5) ∂W/∂E=−∂W/∂F

This equation demonstrates that the optimal option for a manufacturer facing environmental regulations is to make the increase in marginal pollution in production equal to the decrease in marginal pollution from pollution control inputs, i.e., the level of emissions of the manufacturer decreases as the intensity of environmental regulation increases.

From Equations (2), (3) and (5), we derived the following: $\partial W/\;\partial K_{A}>0$, and since $P\left(1-\alpha \right)A^{\prime }f>0$, it is introduced that λ<0.

Substituting this into Equation (3), we obtained W/∂α < 0. This implies that the manufacturer’s pollution emissions will keep decreasing as its investment in pollution control keeps increasing during the production process due to the effects of environmental regulations.

Next, the impact of environmental regulation on urban innovation was examined from a technological innovation perspective.

According to $CI^{\prime }=\left(A,\cdot \right)>0$, then ∂CI/∂A>0, and it can be deduced that:

(6) $$\frac{\partial CI}{\partial A}=\frac{\partial CI}{\partial W}\cdot \frac{\partial W}{\partial A}+\frac{\partial CI}{\partial W}\cdot \frac{\partial W}{\partial E}\cdot \frac{\partial E}{\partial A}>0$$

and because $\frac{\partial W}{\partial A}=\frac{\partial W}{\partial E}\cdot f+\frac{\partial W}{\partial E}\cdot \alpha f$, and $CI=CI_{A}+CI_{E}$, we eventually derived that:

(7) $$\frac{\partial CI}{\partial A}=\left(\frac{\partial CI_{E}}{\partial W}+\frac{\partial CI_{A}}{\partial W}\right)\cdot \left[\frac{\partial W}{\partial F}f\left(1-2\alpha \right)\right]>0$$

where α denotes the intensity of the environmental regulation. From 1−2α>0, ∂W/∂F>0, ∂W/∂E<0, we obtained ∂W/∂α<0.

From Equation (7), it can be deduced that when 0.5>α>0, then $\partial CI_{A}/\partial W>0$. This means that when the level of environmental regulation faced by enterprises is low, with the increase in the intensity of environmental regulation, the emissions of enterprises will decline. However, this will also lead to a decline in technological innovation in enterprise production, which is ultimately detrimental to the improvement of the level of urban innovation. When α>0.5 and tends to 1, $\frac{\partial W}{\partial F}f\left(1-2\alpha \right)<0$ and $\partial CI_{E}/\partial W$ tends to 0, we obtain $\partial CI_{A}/\partial W<0$. At this time, the improvement of technological innovation in enterprises is negatively correlated with pollution emissions. This suggests that the higher the intensity of environmental regulation, the lower the emissions of enterprises, which will increase the technological innovation in enterprise production, leading to a further rise in the level of urban innovation. Therefore, it can be deduced that the effect/impact of the level of environmental regulation on urban innovation is not unique, and further empirical test analysis is required.

## 4. Empirical Design and Analysis

### 4.1. Data Sources and Variable Descriptions

The main sources of panel data for the 281 prefecture-level cities for the period from 2003–2016 are the China Urban Statistical Yearbook, the China City and Industry Innovation Report 2017, the CSMAR database, the WIND database, and www.zhuanli.com (accessed on 24 March 2020).

The explained variable, the urban innovation index (innovation), was measured using the urban innovation index, which is currently a more standardised indicator for measuring innovation capacity at the city level, in addition to patent data. This study also used the patent grant numbers data from www.zhuanli.com (accessed on 24 March 2020) for robustness testing.

The core explanatory variable, i.e., environmental regulation intensity (ERS), was obtained by measuring five indicators using the entropy method with reference to Wang [17]. These five indicators include the sulphur dioxide removal rate, soot removal rate, comprehensive utilisation rate of industrial solid waste, domestic wastewater treatment rate, and domestic waste harmless treatment rate. The specific treatment methods are listed below:

(1) Raw data standardisation.

Positive indicator: $x_{ij}^{\prime }=\left(x_{ij}-\bar{x}\right)/s_{j}$ Reverse indicator: $x_{ij}^{\prime }=\left(\bar{x}-x_{ij}\right)/s_{j}$ where $x_{ij}$ indicates the raw data of the jth indicator of the ith city, $x_{ij}^{\prime }$ represents the standardised indicator values and $\bar{x}$ and $s_{j}$ denote the mean and standard deviation of the jth indicators, respectively. As there were negative values in the standardised data and the entropy method requires logarithmic processing, the standardised data were thus converted to positive values by adding the following constants: $Z_{ij}=x_{ij}^{\prime }+A$

(2) Isomorphism of the indicators and calculating the proportion ($p_{ij}$) of the ith city in the jth indicator ($p_{ij}$)

$$p_{ij}=\frac{Z_{ij}}{\sum_{i=1}^{n}Z_{ij}}\left(i=1,2,\ldots ,281;j=1,2,\ldots ,5\right)$$

(3) Calculation of the entropy value ($e_{j}$) of the jth indicator:

$$e_{j}=-k\sum_{i=1}^{n}p_{ij}\ln\left(p_{ij}\right),\;\mathrm{where}\;k=\frac{1}{\ln\left(n\right)},\;e_{j}\geq 0$$

(4) Calculation of the differentiation factor ($g_{j}$) of the jth indicator: $g_{j}=1-e_{j}$

(5) Normalising the coefficient of variation and calculating the weights ($w_{j}$) of the jth indicator: $w_{j}=g_{j}/\overset{m}{\underset{j=1}{\sum }}\;g_{j}\;\left(j=1,2,\ldots ,m\right)$

(6) Calculation of the environmental regulation intensity ($ERS_{j}$) of the ith city: $ERS_{j}=\overset{m}{\underset{j=1}{\sum }}w_{j}p_{ij}$

Referring to related studies for other control variables, the ratio of secondary and tertiary industries in cities was selected for measuring industrial structure (Industry). The proportion of loan balance in the gross domestic product (GDP) was used to measure financial development (Finde). The natural rate of population growth was used to measure the population growth rate (Growth). The natural logarithm of population size was used to measure the population structure (Lnpeosize). The proportion of financial spending on science and education was used for measuring regional government behaviour (Sciedu), and the Log GDP for measuring regional economic development (LnGDP), among others. These were all control variables in this study. To reduce the estimation bias caused by heteroskedasticity, the standard errors of clustering to the city level were used in this study.

The explained, explanatory, and control variables mentioned above were set up as listed in Table 1. Descriptive statistics of variables are shown in Table 2.

### 4.2. Empirical Regression Results

(1) Baseline regression model

To examine how environmental regulation intensity affects urban innovation capacity, a benchmark regression model was set in this study as follows:

(8) $$Innovation_{it}=\alpha +\beta_{1}ERS_{it}+\underset{j}{\sum }\beta_{j}control_{it}+\mu_{i}+\gamma_{t}+\varepsilon_{it}$$

where Innovation denotes the explanatory variables, ERS as the environmental regulation intensity, $\mu_{i}$ denotes the city fixed effect, $\gamma_{t}$ is the year fixed effect and controlit denotes the control variable and was set as Table 1.

Table 3 presents the baseline regression results. Column (1) denotes results without control variables and fixed effects, Column (2) denotes results without fixed effects and Column (3) represents results having control variables and city fixed effects, while Column (4) denotes results with control variables and city, as well as time fixed effects. The regression results indicate the negative coefficients of environmental regulation intensity and the negative correlation between environmental regulation intensity and China’s urban innovation capacity over the sample period. This result suggests that while intensive environmental regulation may be beneficial to energy conservation and emission reduction at present, but is detrimental to the enhancement of urban innovation capacity. This, in turn, may undermine the ability of cities to achieve long-term energy conservation and emission reduction through innovation over time. Moreover, when two-way city and time fixed effects are added, the coefficient signs of control variables like industrial structure and economic development change significantly. This suggests that heterogeneity may still exist in the way environmental regulation affects the innovation capacity of regional cities and that further mechanism analysis is needed. Additionally, the Porter hypothesis suggests that environmental regulation may enable firms to avoid the cost effects of environmental regulation by promoting innovation for high-quality development.

(2) Robustness test

① The impact of price fluctuations

In this paper, we used the GDP deflator index to convert cities’ GDP data into the constant price based on 2003, which can eliminate the influence of price factors on the regression results and then test the robustness of the baseline regression result. The result report is shown in Table 4. It can be seen that the result after price treatment is still consistent with the baseline regression result, indicating that our baseline regression result is still robust.

② Regional heterogeneity regression test

China’s urban innovation capacity is characterised by a clear regional development imbalance. In particular, provincial capitals and municipalities boast salient advantages in terms of innovation, as they are able to quickly circumvent the adverse effects of environmental regulation through their innovation activities involving innovation funds and talents. However, other small and medium-sized cities may not have such conditions. Thus, the inclusion of provincial capitals and municipalities in the full-sample regression may raise a sample selectivity bias. Hence, the baseline model was reassessed after excluding provincial capitals and municipalities, with results presented in Table 5, and revealed that the environmental regulation intensity still negatively correlated to the innovation capacity of cities, indicating a still robust baseline regression result.

In addition, considering the regional differences in environmental regulations, this paper added more regional heterogeneity regression tests. We made a supplementary investigation on the heterogeneity of economic development and fixed investment in 281 prefecture-level cities by taking the annual mean as the dividing standard. The regression results are shown in Table 6. According to the results in Columns (2) and (4) of Table 6, there is a significant negative correlation between environmental regulation and urban innovation capability in areas with poor economic development and less fixed investment, which indicates that the enhancement of environmental regulation has a more inhibitory effect on the innovation capability of backward areas, while the inhibitory effect on the innovation capability of developed areas is not significant. The possible reasons for this result are as follows: due to the lack of sufficient innovation methods in less developed areas, with the increase of environmental regulation, it is more likely to increase the business pressure of enterprises and reduce the innovation input of enterprises, thus inhibiting the improvement of urban innovation ability. On the contrary, areas with better economic development have a variety of means to avoid environmental regulations, which is more apt to delay the negative impact of environmental regulations on cities’ innovation ability. Moreover, in order to investigate the heterogeneity of urban innovation, we conducted a grouping regression to investigate the difference between knowledge-intensive cities and non-knowledge-intensive cities. Since China began to issue the national innovative City construction list in 2008, the cities on the construction list have had significant advantages in human capital, technological innovation and other aspects. Therefore, this paper took the cities in the list as knowledge-intensive cities, while those not in the list as non-knowledge-intensive cities, andmade regression estimations respectively. The regression results are reported in Columns (5) and (6) of Table 6. It can be found that in non-knowledge-intensive cities, the impact of environmental regulation is significantly negative, while in knowledge-intensive cities, the impact is not significant. This indicated that the increase in environmental regulation intensity has a more significant inhibitory effect on non-knowledge-intensive cities. In addition, since non-knowledge-intensive cities still occupy most of the samples, environmental regulation policies overall still have an inhibiting effect on urban innovation ability.

③ Replacement of urban innovation index

To avoid regression estimation bias due to the indicator measure, the core explained variable in the benchmark regression in the previous section, the urban innovation index (Innovation), was replaced. Patents are an effective indicator of innovation levels and can reflect the level of innovation output of a city. Thus, this study conducted robustness testing by replacing the urban innovation index with the number of patents granted (Patent) as obtained from www.zhuanli.com (accessed on 24 March 2020) for each city as the explained variable (Table 7), and proved the consistency with the baseline regression result, demonstrating the robust regression results in this study.

According to the green patent list and the international classification code provided by the World Intellectual Property Organization (WIPO), we summed up green patent data at the city level and divided them into two parts: green patent application and green patent authorisation. According to the regression results in Table 8, the impact of environmental regulation on green patents is significantly negative at the level of 1% for both green patent applications and green patent grants. This means that environmental regulation policies will lead to the decline of green innovation patents. This is consistent with the original regression results, indicating that the original conclusion has good robustness.

④ Endogenous problem

Additionally, there may exist endogeneity between environmental regulation and urban innovation, as the more innovative a city is, the more likely it is to reduce pollution emissions through innovation, thus rendering it unnecessary for the city to reduce pollution emissions through environmental regulation policies. Thus, a certain reciprocal causality exists. With this in mind, a two-stage least squares analysis of the baseline model was conducted, with air circulation coefficients as instrumental variables. The main considerations for selecting instrumental variables were as follows. First, there exists no theoretical relationship between the air circulation coefficient and the innovation capacity of cities, which satisfies the exogeneity requirement of the instrumental variable. Second, air circulation coefficients correlated to environmental regulation by directly determining the level of air pollution dissipation. Specifically, when at a uniform level of pollution, cities with better air circulation assume that as pollution dissipates quickly, they do not need to adopt stricter environmental regulation policies. However, cities with lower air mobility need to increase their level of environmental regulation to control pollution emissions. Hence, when the air circulation coefficient is higher, the regional environmental regulation policy is theoretically weaker, thus satisfying the exogeneity requirement of instrumental variables. Table 9 presented the endogeneity test with the inclusion of the instrumental variable of air circulation coefficient. The findings of this study remained robust. Moreover, this study conducted a weak instrumental variables test, considering the issue of possible weak instrumental variables between the instrumental variables and environmental regulation. As deduced from the weak instrumental variables test, the F-values (F-weak) in the first stage were all beyond the empirical value of 10, suggesting the absence of weak instrumental variables. Additionally, the regression results for the instrumental variables indicated the conclusions of the current study remained valid following the endogeneity issue addressed.

⑤ Different kinds of environmental regulation policies

In order to reflect the heterogeneity of different environmental regulation policies, we considered two pilot environmental policies as representatives of market-oriented and command-and-control environmental regulations and adopted the difference-in-differences (DID) model to study this problem.

We use carbon emission trading pilot cities as a proxy for market-oriented environmental regulation. This is a Chinese policy launched in 2011, which is similar to the European Union Emissions Trading System (EU-ETS) and aims to achieve optimal economic output with minimal environmental costs. We took the pilot city as the experimental group and the non-pilot city as the control group and designed a DID model as model (9), in which the POL variable was a dummy variable. When the city was included in the list of pilot cities, it was set as 1; otherwise, it was set as 0, and the remaining variables remained unchanged. We focused on the regression coefficient and significance of POL. The regression results are reported in Table 10. Column (1) is listed as the two-way fixed effect regression results without control variables, and Columns (2)–(4) are listed as the regression results of the year fixed effect and city fixed effect gradually added after the addition of control variables. It can be seen that the coefficient of POL has been significantly negative at the level of 1%, which is consistent with the baseline regression results of this paper. It shows that market-oriented environmental regulation can also harm the improvement of urban innovation levels.

(9) $$Innovation_{it}=\alpha +\beta_{1}POL_{it}+\underset{j}{\sum }\beta_{j}\;control_{it}+\mu_{i}+\gamma_{t}+\varepsilon_{it}$$

Secondly, we adopt the Air pollution Prevention and Control Action Plan policy as the representative of command-controlled environmental regulation. The Air Pollution Prevention and Control Action Plan is a policy initiated by China in 2013. It is issued by the State Council on the air pollution prevention and control Action Plan, which makes mandatory requirements for the reduction of the concentration of inhalable particulate matter in different regions of the country. Therefore, it can be used as a representative of command-controlled environmental regulation policy. We took the pilot city as the experimental group and the non-pilot city as the control group and designed a DID model as model (10), in which the variable of AIR was a dummy variable. When the city was included in the list of pilot cities, it was set as 1; otherwise, it was set as 0, and the remaining variables remained unchanged. We focused on the regression coefficient and significance of AIR. The regression results are reported in Table 11. Column (1) is listed as the two-way fixed effect regression result without control variables, Columns (2)–(4) are listed as the regression results of the year fixed effect and city fixed effect gradually added after the addition of control variables. It can be found that the coefficient of AIR is always significantly negative at the level of 1%, which is consistent with the benchmark regression result of this paper. It shows that command-and-control environmental regulation will also damage the improvement of urban innovation levels.

(10) $$Innovation_{it}=\alpha +\beta_{1}AIR_{it}+\underset{j}{\sum }\beta_{j}\;control_{it}+\mu_{i}+\gamma_{t}+\varepsilon_{it}$$

### 4.3. Analysis of the Impact Mechanisms

Regarding the previous baseline regression findings, we concluded that the environmental regulation intensity was strongly negatively related to the innovation capacity of cities during the sample period. We believe that there are two potential mechanisms: government subsidy and enterprise operation. Firstly, with the increase of environmental regulations, the decline in business efficiency of enterprises in the short term will bring a significant decline in local fiscal revenue, and the local government may be forced to reduce the subsidy support for enterprises’ innovation, which will bring a restraining effect on urban innovation ability. Secondly, since most Chinese manufacturing enterprises are still in the transition stage from extensive production to efficient production, blindly strengthening environmental regulation intensity is easy to lead to the decline of local manufacturing output and obstacles to technology research, thus restricting the improvement of urban innovation ability.

Thus, we then clarified the specific mechanism of environmental regulations in affecting urban innovation capacity by constructing the corresponding mediating effect model with two mediating variables: regional fiscal revenue and regional manufacturing output. Drawing on the practice of Xu and Liu [18], the mediating effect was verified by investigating the regression coefficients of a recursive simultaneous equation using a stepwise approach. Taking regional revenue (REV) as a mediating variable, the following test model was constructed.

(11) $$Innovation_{it}=\alpha +\beta_{1}ERS_{it}+\underset{j}{\sum }\beta_{j}\;control_{it}+\mu_{i}+\gamma_{t}+\varepsilon_{it}$$

(12) $$REV_{it}=\alpha_{2}+\delta_{1}ERS_{it}+\underset{j}{\sum }\delta_{j}\;control_{it}+\mu_{i}+\gamma_{t}+\varepsilon_{it}$$

(13) $$Innovation_{it}=\alpha_{3}+\omega_{1}ERS_{it}+\omega_{2}REV_{it}+\underset{j}{\sum }\omega_{j}\;control_{it}+\mu_{i}+\gamma_{t}+\varepsilon_{it}$$

We tested, in turn, the coefficients $\beta_{1}$ of the stepwise model (11), $\delta_{1}$ of model (12) and $\omega_{2}$ of model (13). If all three coefficients were significant, the mediating effect of REV was significant. This indicated that environmental regulation would influence the explained variable urban innovation capacity through the mediating variable REV, with a mediating effect size of $\delta_{1}$ × $\omega_{2}$. The regression results of regional fiscal revenue are presented in Columns (1)–(4) of Table 12. The mediating effect of regional fiscal revenue was $\delta_{1}$ × $\omega_{2}$, which was significantly negative. This indicated that environmental regulation negatively influenced urban innovation capacity through the mediating effect of regional fiscal revenue. Specifically, Column (2) in Table 12 proved the negative relationship between environmental regulation and regional fiscal revenue. Concurrently, Column (3) of Table 12 showed that an increase in fiscal revenue could enhance the city’s innovation capacity. Ultimately, enhanced environmental regulation will bring reduced regional fiscal revenue, which in turn will lead to a decrease in local government support for enterprise innovation policies and, ultimately, a decrease in the urban innovation capacity. These findings are consistent with previous theoretical explanations.

Similarly, regional manufacturing output was also adopted as the mediating variable investigating how environmental regulation contributes to the innovation capacity of cities. We found that the mediating effect of regional manufacturing output was also significant and had a negative coefficient. This indicated that environmental regulations markedly dampened urban innovation capacity through the mediating effect of manufacturing output. Specifically, as can be deduced from Column (5) of Table 12, environmental regulation is significantly negatively related to regional manufacturing output, while Column (6) of Table 12 shows that manufacturing output is positively related to urban innovation capacity. In summary, increased environmental regulation leads to a certain degree of decline in regional manufacturing output, which may constrain firms’ R&D and innovation behaviour, and ultimately lead to a decline in regional urban innovation capacity, validating the relevant explanations presented in the previous section.

To further examine the effect of mediating variables, we have added the interaction term between fiscal revenue and environmental regulations, as well as the interaction term between manufacturing output value and environmental regulations, to model Equation (8), which will be used as a supplement for the robustness test. After adding interaction items into model Equation (8) separately, the new regression results are shown in Table 13. It can be found that no matter the interaction term between fiscal revenue and environmental regulation, or the interaction term between industrial output and environmental regulation, are all significantly negative. This means that environmental regulation policies really have an impact on urban innovation ability through the two factors of regional fiscal revenue and industrial output value. Meanwhile, the higher the regional fiscal revenue and industrial output value, the more significant the inhibition effect of environmental regulation on regional innovation ability. For local governments in China, the negative effects brought by environmental regulation policies will temporarily outweigh the positive effects, which is also an issue that government departments need to consider further.

However, although environmental regulation may inhibit innovation in the short term, there may exist a compensatory effect of innovation; with appropriate environmental regulation incentives, firms may also increase their R&D and innovation, boost energy efficiency and emission reduction and improve their innovation and competitiveness. This finding may also apply to the association between environmental regulation and urban innovation; if regions are able to provide attractive incentives during the environmental regulation process, they may drive business involvement in technological innovation, thus promoting the city’s ability to innovate. Thus, this study further examined the moderating effect of regional economic development and environmental investment on the relationship between environmental regulation and urban innovation capacity by cross-multiplying regional gross national product per capita (GDPPER) and regional environmental investment in pollution control (ENIVES) with the environmental regulation intensity, respectively (Table 14). The coefficients of both the cross multiplier of environmental investment and that of GDP per capita were distinctly positive. Concurrently, the environmental regulation’s coefficient remained apparently negative, but its magnitude was significantly reduced. This indicated that as the regional economy expanded, the inhibitory influence of environmental regulation regulating urban innovation capacity could be effectively mitigated by increasing the positive regulation incentive. Moreover, when the government increases the environmental investment in pollution control, it can somewhat reduce the detrimental influence of environmental regulation on innovation.

### 4.4. Further Discussion

As mentioned earlier, environmental regulation and innovation have a non-linear connection. At lower environmental regulation intensity, it is unnecessary to innovate for environmental protection due to the low expenditure on circumventing environmental regulation. However, the adoption of other methods to circumvent environmental regulation will crowd out investment in innovation to a certain extent. In such cases, environmental regulation can further reduce innovation, whereas as its intensity surpasses a certain level, it becomes challenging to circumvent environmental regulation by other means. Thus, firms may be forced to get around the negative consequences of environmental regulation via innovation. Hence, there may exist a non-linear U-shape relationship between environmental regulation and urban innovation. The current study analysed this issue deeply.

To examine this issue, the squared environmental regulation was introduced in the model, which was designed as follows:

(14) $$innovation_{it}=\alpha +\beta_{1}ERS_{it}+\beta_{2}ERS_{it}^{2}+\underset{j}{\sum }\beta_{j}\;control_{it}+\mu_{i}+\gamma_{t}+\varepsilon_{it\;}$$

If non-linear characteristics existed, then the inflection point at which environmental regulation affects urban innovation was calculated as follows:

(15) $$Inflection\;Point=-\frac{\beta_{1}}{2\beta_{2}}$$

Table 15 reports the regression results for model (14). In the full sample, the squared environmental regulation was remarkably positive, while the first-order term coefficient was obviously negative, indicating the validation of the non-linear characteristic of the U-shape. The calculated inflection points at approximately 0.7 could turn the impact of environmental regulation from negative to positive. Heterogeneity existed in the results for the above three regions. The second-order term coefficient for environmental regulation was insignificant in the eastern region. Conversely, the central and western regions exhibited a non-linear U-shaped characteristic, with an inflection point at approximately 0.6. We think there may be several reasons for this phenomenon. Firstly, the eastern region, as an economically developed region, has been attaching more and more importance to the development of a green economy in recent years, and the intensity of environmental regulations has been increasing year by year. However, local enterprises are generally still in the transition of technological innovation and cannot meet the requirements of environmental regulations in the short term. As a result, environmental regulation has led to the increase of pollution treatment costs and the deterioration of the business environment; innovation behaviour will be significantly inhibited accordingly. Secondly, due to the underdeveloped economy, the central and western regions often enjoy various industrial support policies and capital subsidies from different level governments. This can effectively reduce the problem of rising costs caused by increasing R&D investment. Due to a higher degree of marketisation and fewer government subsidies for production in the eastern region, based on cost-benefit analysis, local enterprises’ operation strategies will be more cautious. Especially in the face of increased environmental regulation, enterprises are more inclined to maintain the stability of daily operation funds, thus reducing R&D investment.

## 5. Conclusions

Porter’s hypothesis has prompted scholars to address the micro effects of environmental regulations from a firm’s perspective. The current research examined the micro effects and mechanisms affecting the innovation capacity of cities by constructing theoretical models and empirical tests. We discovered that environmental regulation substantially negatively correlated to China’s urban innovation capacity during the sample period, and its increasing intensity could dampen China’s urban innovation capacity. Concurrently, this inhibitory effect was mainly transmitted through two mediating variables: lower regional fiscal revenue and reduced manufacturing output. Increased regional economic development helps to bring positive incentives for environmental regulation, thus somewhat mitigating the inhibiting effect of increased environmental regulation on urban innovation capacity. Additionally, there may exist a non-linear U-shape relationship between environmental regulation and urban innovation; the sufficiently high intensity of environmental regulation will force firms to innovate and circumvent the drawbacks of environmental regulation.

Thus, the following issues should be considered during the policy development of environmental regulation in China. Firstly, as China is in the transition from extensive development to high-quality development, the negative effects of environmental regulation policies will temporarily outweigh the positive effects. Therefore, it is necessary to be alert to the negative spillover effects of environmental regulation on technological innovation. Secondly, local governments should be cautious about the adverse effects brought about by environmental regulation at the initial stage and implement reasonable emission policies to enable enterprises to survive the period of loss resulting from environmental regulation to help enhance innovation in companies and cities. Thirdly, local governments must rationally judge the characteristics of environmental regulation inflection points on their own circumstances and formulate corresponding environmental regulation policies. Fourthly, in more economically developed regions, local governments may consider supporting innovative firms with financial subsidies or tax concessions to enter the innovation dividend period of environmental regulation. Fifthly, for economically less-developed regions, due to the lack of sufficient innovative means, the intensity of environmental regulation by the local government should not be too high, which can avoid directly increasing the pressure on business operations. It will be helpful to mitigate the negative impact of environmental regulation on local economic development. Future studies may focus on a comparison between developing and developed regions in China to find the differences in innovation levels and remedies to boost innovation through environmental regulations in the deprived areas.

## Figures and Tables

**Table 1:** Setting of the main variables.

	Name	Definition	Properties
Explained variables	Innovation	Innovation index, number of patents	Innovation indicators
Explanatory variables	ERS	Environmental regulation intensity score	Environmental regulation variables
Control variables	Industry	Ratio of the secondary and tertiary industries	Urban Characteristics
	LnGDP	Logarithm of the gross national product	
	Finde	Financial development	
	Growth	Natural population growth rate	
	Lnpeosize	Population size in logarithms	
	Sciedu	Proportion of financial spending on science and education	

**Table 2:** Variables description.

Variable	N	Mean	Sd	Min	Max
Innovation	3929	7.053	39.03	0	1100
ERS	3929	0.652	0.157	0.169	0.978
Industry	3926	1.467	0.783	0.106	10.60
Finde	3646	0.824	1.557	0.0753	90.16
Growth	3911	5.952	4.843	−8.900	40.78
Lnpeosize	3929	5.856	0.693	2.796	8.129
Sciedu	3926	0.198	0.047	0.0158	0.497
LnGDP	3927	6.719	1.063	3.459	10.25

**Table 3:** Baseline regression results.

	(1)	(2)	(3)	(4)
	Innovation	Innovation	Innovation	Innovation
ERS	−15.0208 **	−25.7736 ***	−24.8920 ***	−18.5348 ***
	(−2.54)	(−4.36)	(−3.97)	(−3.08)
Industry		−7.1888 ***	−7.2840 ***	2.3303 **
		(−6.43)	(−6.74)	(2.41)
Finde		1.8014 **	1.8023 **	−0.6837 ***
		(1.99)	(2.26)	(−2.94)
Growth		0.3309	0.4262 *	0.3173 ***
		(1.59)	(1.90)	(2.89)
Lnpeosize		−8.3218 ***	−11.1270 ***	97.8779 ***
		(−4.70)	(−5.19)	(2.65)
Sciedu		−2.6257	9.7068	121.5947 ***
		(−0.27)	(0.99)	(5.35)
LnGDP		20.1563 ***	23.2931 ***	−15.9533 ***
		(7.95)	(7.87)	(−3.59)
_cons	343.8323 ***	−56.0059 ***	−52.5999 ***	−2.1 × 10^2^
	(3.99)	(−7.83)	(−7.59)	(−0.84)
City	YES	NO	YES	YES
Year	YES	NO	NO	YES
N	3929	3624	3624	3624
R-sq	0.540	0.186	0.199	0.595

Note: ***, ** and * represent the significance at the 1%, 5% and 10% levels, respectively. The *t*-values are in parentheses.

**Table 4:** Robustness test results of GDP constant price.

	(1)	(2)	(3)	(4)
	Innovation	Innovation	Innovation	Innovation
ERS	−15.0208 **	−18.2479 ***	−22.3618 ***	−17.3725 ***
	(−2.54)	(−3.61)	(−3.88)	(−3.53)
Industry		−7.9571 ***	−6.9628 ***	1.9263 **
		(−6.71)	(−6.72)	(2.43)
Finde		1.8835 **	1.8521 **	−0.5264 ***
		(2.20)	(2.43)	(−3.12)
Growth		0.4635 **	0.7811 *	0.4281 ***
		(2.13)	(1.92)	(2.99)
Lnpeosize		−11.0072 ***	−10.2381 ***	96.2313 ***
		(−5.29)	(−4.92)	(3.78)
Sciedu		3.3873	9.1273	123.234 ***
		(0.34)	(0.12)	(4.72)
LnGDP		22.7693 ***	24.2341 ***	−16.1926 ***
		(7.98)	(6.92)	(−4.21)
_cons	343.8323 ***	−54.4965 ***	−52.2381 ***	−2.1 × 10^2^
	(3.99)	(−7.77)	(−6.46)	(−0.75)
City	YES	NO	YES	YES
Year	YES	NO	NO	YES
N	3929	3624	3624	3624
R-sq	0.540	0.192	0.199	0.595

Note: ***, ** and * represent the significance at the 1%, 5% and 10% levels, respectively. The *t*-values are in parentheses.

**Table 5:** Robustness test results excluding provincial capitals and municipalities.

	(1)	(2)	(3)	(4)
	Innovation	Innovation	Innovation	Innovation
ERS	−12.0634 **	−12.3951 **	−10.3517 *	−13.4969 **
	(−2.25)	(−2.31)	(−1.95)	(−2.40)
Industry		−3.7860 ***	−4.3018 ***	0.3077
		(−4.41)	(−4.21)	(0.52)
Finde		0.9207 ***	1.0316 ***	−0.2241 *
		(2.65)	(2.86)	(−1.72)
Growth		0.4800 **	0.5767 **	0.1030
		(2.21)	(2.34)	(1.31)
Lnpeosize		−7.5658 ***	−9.5432 ***	96.5647 **
		(−4.08)	(−4.07)	(2.23)
Sciedu		2.7756	3.5675	55.9606 ***
		(0.34)	(0.41)	(3.10)
LnGDP		11.0440 ***	13.6805 ***	−7.9811 ***
		(4.75)	(4.58)	(−3.07)
_cons	7.5912 **	−16.3936 ***	−15.6138 ***	−5.8 × 10^2^ **
	(2.31)	(−8.71)	(−8.12)	(−2.15)
City	YES	NO	YES	YES
Year	YES	NO	NO	YES
N	3509	3235	3235	3235
R-sq	0.494	0.140	0.160	0.576

Note: ***, ** and * represent the significance at the 1%, 5% and 10% levels, respectively. The *t*-values are in parentheses.

**Table 6:** Robustness test results of regional economic development, fixed investment and knowledge-intensive city.

	(1)	(2)	(3)	(4)	(5)	(6)
	High_GDP	Low_GDP	High_FIX	Low_FIX	Knowledge-Intensive City	Non-Knowledge-Intensive City
ERS	−23.1933	−1.0307 ***	−25.7282	−0.6652 **	−50.8068	−2.1908 ***
	(−0.72)	(−3.85)	(−0.86)	(−2.52)	(−0.58)	(−3.37)
Industry	17.0594 ***	0.1807 **	17.6586 ***	0.2892 ***	122.8785 **	0.0423
	(3.17)	(2.32)	(2.80)	(3.87)	(2.15)	(0.27)
Finde	−8.9773	0.3993 *	−3.0338 ***	0.3195 *	−35.3804	0.0725
	(−0.99)	(1.90)	(−3.51)	(1.84)	(−1.40)	(1.63)
Growth	1.0362 *	0.0217 ***	0.9156 *	0.0212 ***	1.9645	0.0614 ***
	(1.68)	(3.11)	(1.71)	(3.09)	(1.54)	(3.72)
Lnpeosize	190.0496 *	9.2486 ***	165.6567 **	5.2244 ***	365.4422 **	6.2423
	(1.92)	(4.15)	(2.03)	(4.34)	(2.59)	(1.52)
Sciedu	248.5177 ***	10.0942 ***	256.0448 ***	8.5292 ***	−8.1 × 10^2^ **	30.9878 ***
	(2.88)	(6.48)	(2.91)	(6.67)	(−1.97)	(7.25)
LnGDP	−43.3764 **	−0.0782	−58.8607 ***	−1.1771 ***	−17.9567	−1.9222 ***
	(−2.16)	(−0.24)	(−3.39)	(−3.67)	(−0.38)	(−2.86)
_cons	−6.5 × 10^2^	−51.3693 ***	−3.6 × 10^2^	−22.1732 ***	−1.9 × 10^3^ *	71.7138 **
	(−0.97)	(−3.93)	(−0.69)	(−3.34)	(−1.73)	(2.11)
city	YES	YES	YES	YES	YES	YES
year	YES	YES	YES	YES	YES	YES
N	941	2683	1055	2569	298	3326
R-sq	0.640	0.643	0.634	0.812	0.864	0.736

Note: ***, ** and * represent the significance at the 1%, 5% and 10% levels, respectively. The *t*-values are in parentheses.

**Table 7:** Results of robustness tests after replacing the urban innovation index.

	(1)	(2)	(3)	(4)
	Patent	Patent	Patent	Patent
ERS	−4.2646 ***	−3.8763 ***	−3.0519 ***	−3.8006 ***
	(−5.80)	(−4.80)	(−4.19)	(−5.40)
Industry		−1.7383 ***	−1.8409 ***	−0.2981
		(−8.73)	(−8.76)	(−1.30)
Finde		0.5098 ***	0.5309 ***	0.0923 **
		(7.05)	(8.42)	(2.01)
Growth		0.1099 **	0.1401 ***	0.0949 ***
		(2.56)	(2.97)	(4.47)
Lnpeosize		−3.3814 ***	−4.0289 ***	11.6817
		(−7.23)	(−7.43)	(1.55)
Sciedu		9.8662 ***	11.8644 ***	32.5693 ***
		(3.80)	(4.36)	(5.53)
LnGDP		5.8164 ***	6.5426 ***	−2.2969 ***
		(11.78)	(11.33)	(−2.65)
_cons	5.7103 ***	−14.9866 ***	−14.3915 ***	−65.7841
	(7.56)	(−13.31)	(−13.22)	(−1.37)
City	YES	NO	YES	YES
Year	YES	NO	NO	YES
N	3047	3024	3024	3024
R-sq	0.734	0.340	0.358	0.757

Note: *** and ** represent the significance at the 1% and 5% levels, respectively. The *t*-values are in parentheses.

**Table 8:** Results of robustness tests after dividing green patent into two parts: green patent application and green patent authorisation.

	(1)	(2)	(3)	(4)	(5)	(6)	(7)	(8)
	Green Patent Application				Green Patent Authorisation			
	GreenIno1	GreenIno1	GreenIno1	GreenIno1	GreenIno2	GreenIno2	GreenIno2	GreenIno2
ERS	−6.4662 ***	−0.9222 ***	−0.7430 ***	−0.5525 ***	−5.5415 ***	−0.3178 ***	−0.4506 ***	−0.3098 ***
	(−43.33)	(−8.27)	(−6.44)	(−4.80)	(−39.34)	(−2.86)	(−3.92)	(−2.75)
Industry		−0.2646 ***	−0.2368 ***	−0.2940 ***		−0.2368 ***	−0.2480 ***	−0.3111 ***
		(−12.52)	(−11.24)	(−14.23)		(−11.01)	(−11.57)	(−15.19)
Finde		0.1210 ***	0.1175 ***	0.1104 ***		0.1254 ***	0.1245 ***	0.1136 ***
		(3.23)	(3.22)	(4.73)		(2.92)	(3.06)	(4.55)
Growth		−0.0010	−0.0043	0.0013		−0.0037	−0.0026	0.0029
		(−0.37)	(−1.45)	(0.40)		(−1.40)	(−0.93)	(0.91)
Lnpeosize		−0.3069 ***	−0.2883 ***	−0.4833 ***		−0.2969 ***	−0.3588 ***	−0.5278 ***
		(−10.62)	(−9.41)	(−14.13)		(−9.93)	(−11.38)	(−15.32)
Sciedu		−1.1194 ***	−0.8162 ***	−0.5861		−1.1134 ***	−0.7788 **	−0.9079 **
		(−3.71)	(−2.62)	(−1.60)		(−3.59)	(−2.46)	(−2.47)
LnGDP		1.5265 ***	1.5159 ***	1.7063 ***		1.4555 ***	1.5170 ***	1.7105 ***
		(69.17)	(61.23)	(66.06)		(62.78)	(59.30)	(64.71)
_cons	−1.4687 ***	−5.8220 ***	−5.7905 ***	−4.8744 ***	−1.6625 ***	−5.8496 ***	−5.7679 ***	−4.7252 ***
	(−15.82)	(−40.03)	(−38.57)	(−23.66)	(−19.42)	(−39.16)	(−37.49)	(−22.65)
City	YES	NO	YES	YES	YES	NO	YES	YES
Year	YES	NO	NO	YES	YES	NO	NO	YES
N	3901	3599	3599	3599	3901	3599	3599	3599
R-sq	0.799	0.319	0.803	0.843	0.765	0.170	0.771	0.814

Note: *** and ** represent the significance at the 1% and 5% levels, respectively. The *t*-values are in parentheses.

**Table 9:** Endogeneity test with the inclusion of the instrumental variable (air circulation coefficient).

	(1)	(2)	(3)	(4)
	Innovation	Innovation	Innovation	Innovation
ERS	−17.6497 *	−29.3929 ***	−26.3977 ***	−19.9488 *
	(−1.65)	(−4.38)	(−3.83)	(−1.89)
Industry		−7.2118 ***	−7.2766 ***	2.3639 *
		(−8.80)	(−8.72)	(1.77)
Finde		1.8223 ***	1.8091 ***	−0.6815 *
		(4.62)	(4.61)	(−1.86)
Growth		0.3404 ***	0.4280 ***	0.3239 **
		(2.65)	(3.26)	(2.27)
Lnpeosize		−8.5855 ***	−11.1972 ***	98.1400 ***
		(−6.70)	(−8.41)	(10.28)
Sciedu		−1.8387	9.8361	121.6783 ***
		(−0.13)	(0.67)	(6.90)
LnGDP		20.5667 ***	23.4263 ***	−15.8856 ***
		(20.04)	(21.71)	(−3.69)
_cons	396.2816 ***	−55.0671 ***	−62.2522 ***	−1.7 × 10^2^ **
	(32.27)	(−8.67)	(−8.74)	(−2.27)
City	YES	NO	YES	YES
Year	YES	NO	NO	YES
N	3643	3619	3619	3619
R-sq	0.573	0.186	0.199	0.595
F-weak	27.84	25.21	36.23	26.35

Note: ***, ** and * represent the significance at the 1%, 5% and 10% levels, respectively. The *t*-values are in parentheses.

**Table 10:** DID regression results of market-oriented environmental regulation.

	(1)	(2)	(3)	(4)
Market-Oriented	Innovation	Innovation	Innovation	Innovation
POL	−48.9229 ***	−33.6805 ***	−35.9997 ***	−35.4096 ***
	(−4.91)	(−4.20)	(−4.36)	(−5.64)
Industry		−5.9974 ***	−6.4375 ***	0.9284
		(−6.79)	(−7.19)	(1.18)
Finde		1.7430 *	1.8286 **	−0.4375 **
		(1.74)	(2.01)	(−2.20)
Growth		0.0565	0.1869	0.0858
		(0.31)	(0.95)	(0.88)
Lnpeosize		−4.6313 ***	−8.5713 ***	94.1931 ***
		(−3.50)	(−4.87)	(2.79)
Sciedu		−25.2315 **	−11.0340	112.8472 ***
		(−2.16)	(−1.00)	(5.24)
LnGDP		15.0302 ***	19.3583 ***	−12.3454 ***
		(10.23)	(9.28)	(−3.32)
_cons	4.3632 ***	−57.4117 ***	−54.1978 ***	−2.4 × 10^2^
	(17.39)	(−8.64)	(−8.18)	(−1.02)
City	YES	NO	YES	YES
Year	YES	NO	NO	YES
N	3929	3624	3624	3624
R-sq	0.582	0.215	0.233	0.614

Note: ***, ** and * represent the significance at the 1%, 5% and 10% levels, respectively. The *t*-values are in parentheses.

**Table 11:** DID regression results of command-controlled environmental regulation.

	(1)	(2)	(3)	(4)
Command–Controlled	Innovation	Innovation	Innovation	Innovation
AIR	−16.2614 ***	−14.9303 ***	−7.0667 ***	−7.7409 ***
	(−6.19)	(−4.57)	(−4.89)	(−4.19)
Industry		−7.0636 ***	−7.3537 ***	2.2818 **
		(−6.44)	(−6.71)	(2.39)
Finde		1.6783 *	1.6931 **	−0.6838 ***
		(1.90)	(2.18)	(−2.94)
Growth		0.2704	0.3965 *	0.3241 ***
		(1.30)	(1.80)	(2.93)
Lnpeosize		−6.7099 ***	−9.9680 ***	97.6943 ***
		(−4.14)	(−5.08)	(2.65)
Sciedu		−9.8392	4.1007	121.1801 ***
		(−1.02)	(0.43)	(5.33)
LnGDP		17.5897 ***	21.1076 ***	−16.1490 ***
		(8.67)	(8.37)	(−3.63)
_cons	3.5886 ***	−63.1212 ***	−58.9005 ***	−2.2 × 10^2^
	(10.78)	(−7.88)	(−7.66)	(−0.87)
City	YES	NO	YES	YES
Year	YES	NO	NO	YES
N	3929	3624	3624	3624
R-sq	0.529	0.180	0.195	0.595

Note: ***, ** and * represent the significance at the 1%, 5% and 10% levels, respectively. The *t*-values are in parentheses.

**Table 12:** Mechanisms by which environmental regulation affects the innovation capacity of Chinese cities.

	(1)	(2)	(3)	(4)	(5)	(6)	(7)
	Innovation	REV	Innovation	Innovation	IND	Innovation	Innovation
ERS	−18.5348 ***	−1.0947 ***		−3.6768 **	−2.0583 ***		−14.6193 **
	(−3.08)	(−3.60)		(−2.29)	(−3.61)		(−2.47)
REV			0.1358 ***	0.1357 ***			
			(10.03)	(10.03)			
IND						1.9322 ***	−1.9023 ***
						(6.55)	(−6.46)
Industry	2.3303 **	−0.0910	3.5331 ***	3.5658 ***	7.4853 ***	16.6603 ***	16.5699 ***
	(2.41)	(−1.43)	(5.18)	(5.21)	(12.47)	(5.36)	(5.33)
Finde	−0.6837 ***	0.0133	−0.8683 ***	−0.8641 ***	0.3236 ***	−0.0745	−0.0680
	(−2.94)	(0.83)	(−6.58)	(−6.56)	(4.13)	(−0.38)	(−0.35)
Growth	0.3173 ***	0.0246 ***	−0.0176	−0.0166	−0.0108	0.2937 ***	0.2969 ***
	(2.89)	(4.14)	(−0.34)	(−0.32)	(−0.77)	(2.82)	(2.85)
Lnpeosize	97.8779 ***	3.9291 ***	44.5504 ***	44.5507 ***	−5.1356 **	88.1182 **	88.1082 **
	(2.65)	(2.66)	(2.68)	(2.68)	(−2.28)	(2.57)	(2.57)
Sciedu	121.5947 ***	10.3820 ***	−19.4134	−19.3114	−15.3751 ***	91.9168 ***	92.3459 ***
	(5.35)	(7.23)	(−1.14)	(−1.13)	(−6.01)	(4.71)	(4.74)
LnGDP	−15.9533 ***	0.1233	−17.7765 ***	−17.6274 ***	8.0326 ***	−1.0194	−0.6725
	(−3.59)	(0.41)	(−6.95)	(−6.91)	(5.46)	(−0.27)	(−0.18)
_cons	−2.1 × 10^2^	−5.0168	−1.5 × 10^2^	−1.4 × 10^2^	−11.0899	−2.4 × 10^2^	−2.3 × 10^2^
	(−0.84)	(−0.48)	(−1.22)	(−1.21)	(−0.67)	(−1.00)	(−0.98)
city	YES	YES	YES	YES	YES	YES	YES
year	YES	YES	YES	YES	YES	YES	YES
N	3624	3624	3624	3624	3624	3624	3624
R-sq	0.595	0.793	0.875	0.875	0.945	0.609	0.610

Note: *** and ** represent the significance at the 1% and 5% levels, respectively. The *t*-values are in parentheses.

**Table 13:** Regression results of interaction terms between intermediate variable and environmental regulations.

	(1)	(2)
	Innovation	Innovation
REV × ERS	−6.7466 ***	
	(−3.88)	
REV	19.7336 ***	
	(2.73)	
IND×ERS		−2.4905 ***
		(−4.61)
IND		−0.3617 *
		(−1.75)
ERS	1.7487	109.0976 ***
	(0.34)	(3.89)
Industry	3.6497 ***	15.0186 ***
	(5.20)	(5.26)
Finde	−0.9281 ***	0.0758
	(−6.05)	(0.41)
Growth	−0.0265	0.2791 ***
	(−0.49)	(2.69)
Lnpeosize	43.7520 ***	87.6656 **
	(2.80)	(2.56)
Sciedu	−21.4370	100.4303 ***
	(−1.27)	(5.12)
LnGDP	−18.9218 ***	2.7377
	(−6.27)	(0.75)
_cons	−1.5 × 10^2^	−3.5 × 10^2^
	(−1.33)	(−1.44)
city	YES	YES
year	YES	YES
N	3624	3624
R-sq	0.877	0.615

Note: ***, ** and * represent the significance at the 1%, 5% and 10% levels, respectively. The *t*-values are in parentheses.

**Table 14:** Moderating effects of regional economic development and investment.

	(1)	(2)
	Innovation	Innovation
ENIVES × ERS	3.2339 ***	
	(3.59)	
ENIVES	0.0019	
	(0.52)	
GDPPER×ERS		6.9643 ***
		(3.06)
GDPPER		3.7167 ***
		(3.58)
ERS	−2.0510 **	−2.0686 **
	(2.44)	(−2.40)
Industry	−0.7466 **	10.7979 ***
	(−2.58)	(5.93)
Finde	−2.9813	−1.1922 ***
	(−1.51)	(−3.51)
Growth	0.0167	0.1701 **
	(0.69)	(2.10)
Lnpeosize	21.6479 **	101.6441 ***
	(1.98)	(3.32)
Sciedu	9.7721 **	−40.1351
	(2.15)	(−1.59)
LnGDP	−2.1484 *	−44.3047 ***
	(−1.77)	(−4.47)
_cons	−54.7029	−44.8829
	(−0.70)	(−0.24)
City	YES	YES
Year	YES	YES
N	1055	3624
R-sq	0.619	0.690

Note: ***, ** and * represent the significance at the 1%, 5% and 10% levels, respectively. The *t*-values are in parentheses.

**Table 15:** Non-linear relationship between environmental regulation and the innovation capacity of cities.

	Full Sample	Eastern Region	Central Region	Western Region
ERS	−96.4268 ***	−92.3252 *	−62.3718 ***	−60.8416 ***
	(−4.05)	(−1.65)	(−5.90)	(−4.72)
ERS²	64.8753 ***	44.3182	52.3886 ***	53.2727 ***
	(3.36)	(1.01)	(5.99)	(4.98)
Industry	2.6925 *	6.3187 *	0.3550	0.2932
	(1.93)	(1.85)	(0.35)	(0.51)
Finde	−0.6514 *	−1.1470 *	0.8292	8.6236 ***
	(−1.71)	(−1.82)	(1.26)	(6.34)
Growth	0.3052 **	0.3501	0.1127	0.0837
	(2.05)	(1.11)	(1.45)	(1.08)
Lnpeosize	95.2399 ***	113.7912 ***	−6.4220	25.2700 ***
	(9.55)	(7.15)	(−0.80)	(3.09)
Sciedu	115.6751 ***	183.1929 ***	54.7605 ***	45.7342 ***
	(6.27)	(5.16)	(5.18)	(4.43)
LnGDP	−15.2994 ***	−22.4736 **	10.0248 ***	8.8039 ***
	(−3.42)	(−2.39)	(4.03)	(3.55)
_cons	−1.8 × 10^2^ **	−2.6 × 10^2^ **	−10.6416	−2.3 × 10^2^ ***
	(−2.36)	(−2.03)	(−0.23)	(−4.27)
City	Yes	yes	yes	yes
Year	Yes	yes	yes	yes
N	3624	1765	934	925
R-sq	0.596	0.607	0.628	0.611
Inflection point	0.7		0.6	0.6

Note: ***, ** and * represent the significance at the 1%, 5% and 10% levels, respectively. The *t*-values are in parentheses.

## Data Availability

No datasets were generated or analyzed in this study.
